# Supplementary material for: Impact of bulk-edge coupling on observation of anyonic braiding statistics in quantum Hall interferometers
Source: Nat Commun. 2022 Jan 17;13:344. doi: 10.1038/s41467-022-27958-w (PMC8763912; doi:10.1038/s41467-022-27958-w)
Supplement: Supplementary file 1 — Supplementary Information [file 41467_2022_27958_MOESM1_ESM.pdf]

Supplementary Material for "Impact of bulk-edge coupling on observation of anyonic braiding statistics in quantum Hall interferometers"

SUPPLEMENTAL SECTION 1: REPEATABILITY OF DATA AT $\nu = 1/3$

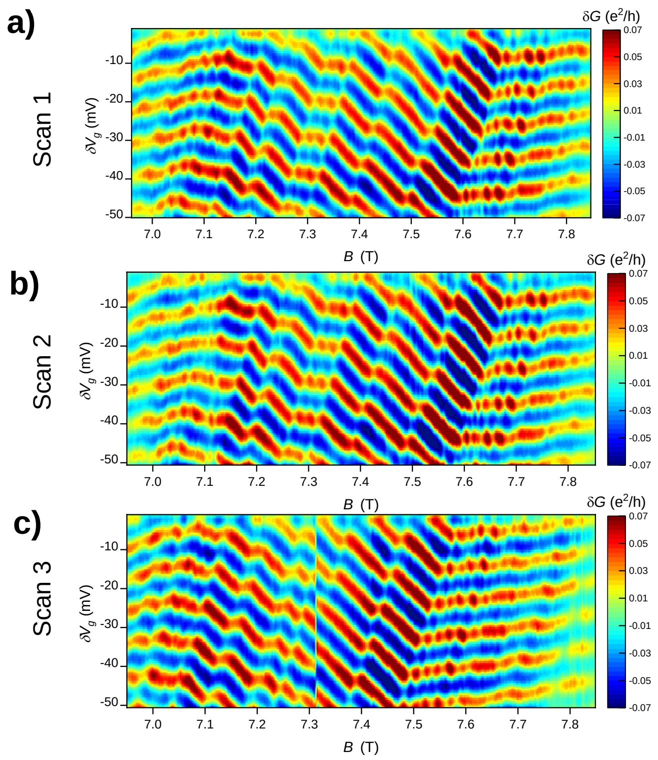

SUPP. FIG. 1. a) 1st scan of conductance versus magnetic field and gate voltage at $\nu = 1/3$. This is the same data presented in the main text. b) 2nd scan over the same magnetic field range using the same paramaters. c) 3rd scan across the same region. In this third scan a clear switching event occurs at approximately 7.3 T.

The behavior observed at $\nu = 1/3$ was found to be repeatable upon subsequent measurements. This is shown in Fig. 1, with panels a, b, and c showing conductance versus gate voltage in three separate scans taken one after the other (the first scan is shown in the main text). The discrete jumps in the central region and behavior in the high and low field region are repeatable in each scan. Each scan takes approximately 3 hours. Notably, in the third scan a switching event is visible at approximately 7.3 T.

Measurements are taken by sweeping gate voltage from the starting value to the final value (from higher to lower voltage) while measuring the conductance, returning the gate voltage to the starting value, then taking a step in magnetic field of 2 mT and repeating the process across the whole magnetic field range. Therefore, changes in the electrostatic potential due to charge noise (which is common in dopeed GaAs/AlGaAs heterostructures) should result in vertical discontinuities in the conductance pattern, such as the one visible in Scan 3. Though we observe occasional jumps like this in our device, they are relatively rare, with only one such jump visible in the approximately 3 hour measurement of Scan 3, and none visible in Scans 1 or 2, which indicates fairly stable device operation. We have used the technique of bias cooling in which a positive bias of +600 mV is applied to the gate when cooling from room temperature, which enables smaller gate voltages to be used and thus reduces charge noise. It is noteworthy that the discrete jumps in phase highlighted in the main text attributed to changes in localized quasiparticle number appear quite different from the switching events associated with charge noise. The discrete jumps have a consistent trend with a positive slope in the magnetic field-gate voltage plane, indicating that they are not an effect due to random charge noise fluctuations.

Additionally, note that both the isolated discrete jumps in phase and the transition to the low and high field regions occur with approximately the same slope in the $B - V_g$ plane of ≈ 0.4 V/T. This supports the idea that the discrete phase jumps and the low/high field shifts in behavior are both the result of changing quasiparticle number. The fact that the slope is positive can be understood from the fact that increasing magnetic field is expected to remove quasiparticles (or add quasiholes), while increasing gate voltage should favor adding quasiparticles (or removing quasiholes). It is also noticeable that the lines of constant phase in the high and low field regions are not perfectly flat, but have a slight positive slope. While zero dependence on magnetic field would be expected if all device parameters were independent of magnetic field, the slight positive slope may suggest that increasing B exerts a small negative effective gate voltage due to the increase in cyclotron energy; similar behavior has been observed in quantum dots in the integer quantum Hall regime [1, 2].

SUPPLEMENTAL SECTION 2: FOURIER TRANSFORMS AT $\nu = 1/3$

2D Fourier transforms from the data in Fig. 1c from the main text are shown in Fig. 2. The transform in Fig. 2a corresponds to the low field region (from 6.95 T to 7.17 T), b corresponds to the central region (7.17 T to 7.65 T), and c corresponds to the high field region (7.65 T to 7.85 T).

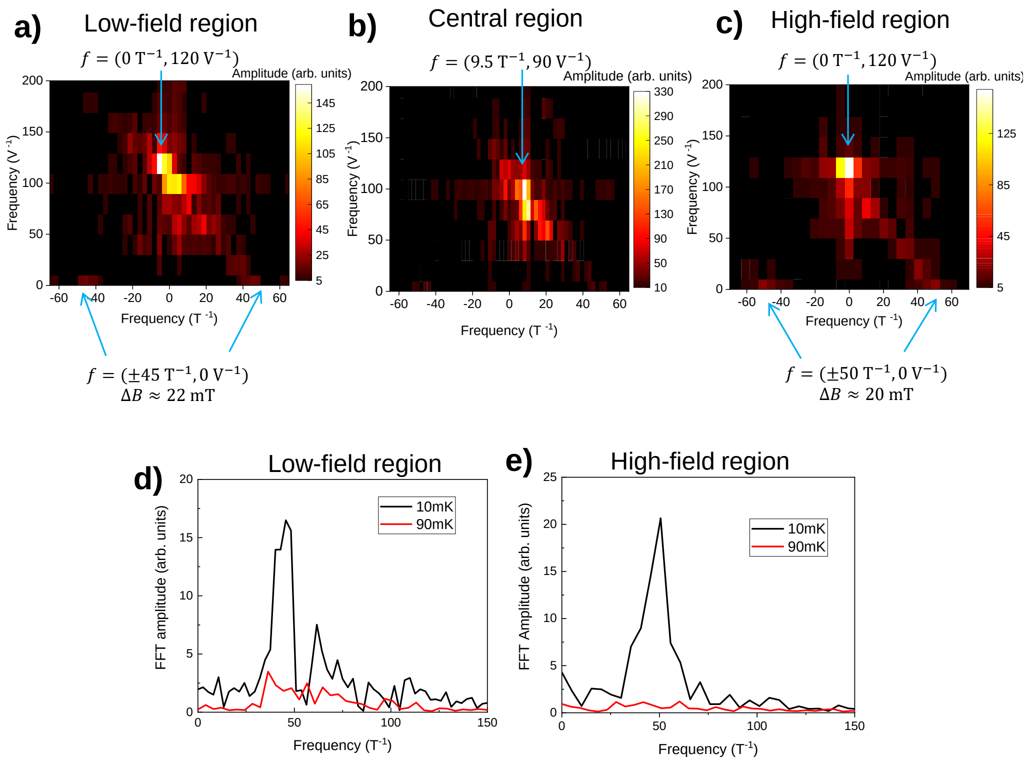

SUPP. FIG. 2. 2D Fourier transforms of the data in the low-field region from Fig. 1c of the main text in a) the low field region, b) the central region, and c) the high field region. d) Line cuts of the FFT amplitude versus magnetic field frequency at zero gate-voltage frequency, at 10 mK (black) and 90 mK (red) in the low-field region. e) Line cuts at zero gate-voltage frequency in the high-field region.

The peak frequencies in the high and low field regions occur at close to 0 magnetic field frequency and 120 V^{-1} gate voltage frequency (corresponding to a period of approximately 8.3 mV). The fact that this peak occurs at zero magnetic field frequency is consistent with expectations for a compressible bulk when μ is outside of the energy gap [11]. In addition to this primary peak, there is a much smaller peak at $\pm 45\ \mathrm{T}^{-1}$ in the low field region and $\pm 50\ \mathrm{T}^{-1}$ in the high field region at 0 gate voltage frequency, corresponding periods of 22 mT and 20 mT. These peaks come from modulations visible in the data (Fig. 1c in the main text), and the periods are close the period of ≈ 20 mT measured for Aharonov-Bohm interference at $\nu = 1$ (in the compressible region at $\nu = 1$ where bulk-edge interaction does not modify the period), indicating that this period corresponds to Φ_0. The Φ_0 period modulations are an expected signature of anyonic braiding statistics when the bulk is compressible [7, 11] resulting from period changes in localized quasiparticle number. The fact that this peak occurs at zero gate voltage frequency may be a coincidence due to the fact that the lever arm connecting the side gates to the bulk, α_{buk}, is approximately half of the lever arm connecting the gate to the edge, α_{edge}. The estimated lever arms for the side gates to the edge is $\alpha_{edge} = 0.074\ \mathrm{mV}^{-1}$, and the lever arm for the edge to the bulk is $\alpha_{bulk} = 0.044\ \mathrm{mV}^{-1}$ based on the $\nu = 1$ interference gate voltage period and the $B = 0$ Coulomb blockade period (here the lever arms represent the number of electrons moved per mV change in gate voltage).

It is noteworthy that these modulations have an oscillation period very close to the $\nu = 1$ Aharonov-Bohm period. This strongly indicates that the effective area of the interferometer does not change significantly between $\nu = 1$ and $\nu = 1/3$.

In principle higher frequency harmonics at multiples of Φ_0 might occur, since the change in phase when the quasiparticle number changes is discrete, which would result in a sawtooth-like conductance pattern rather than sinusoidal oscilations [4]. However, peaks at these higher harmonics are not visible, likely due to thermal smearing which makes the quasiparticle transitions not sharp.

Line cuts of the FFT amplitude vs. magnetic field frequency are shown in Fig. 2d and e for the low and high field regions. The peaks close to the Φ_0 frequency are clearly visible. In red, Fourier transform line cuts at elevated mixing chamber temperature of 90 mK are shown; at this elevated temperature the Φ_0 peaks not visible, consistent with thermal smearing of the quasiparticle number. This reinforces that the Φ_0 modulations

are a higher-order contribution to interference which is quickly suppressed as T increases, while the leading order behavior has no B dependence. Note that the charging energy should set the energy scale which determines the visibility of the Φ_0 modulations in the interference pattern at $\nu = 1/3$. According to Ref. [7] the energy scale for thermal damping of the Φ_0 period is $\frac{(e^*)^2 E_c}{\pi^2}$. Using $E_c = 72$ μeV based on the Coulomb blockade measurements discussed in the main text yields an energy scale of 9.4 mK. Since our dilution refrigerator has a base temperature of approximately 10 mK, it is reasonable that the Φ_0 modulations would be visible for this device at base temperature, but not for larger devices with significantly smaller charging energies or at elevated temperatures.

In the central region the phase evolves primarily due to the Aharonov-Bohm phase, but the isolated discrete jumps also have a noticeable impact on the Fourier spectrum. The peak in the central region occurs at approximately 9.5 T^{-1} and 90 V^{-1}. The magnetic field period is lower than the frequency of 12 T $^{-1}$ that would be predicted based only on the oscillation period of 83 mT in the regions between the phase jumps. The shift in frequency occurs due to the discrete jumps in phase, which increase the spacing between peaks and minima in conductance in the regions where they occur, shifting the Fourier peak to lower frequency.

SUPPLEMENTAL SECTION 3: ESTIMATING BULK-EDGE COUPLING FROM MAGNETIC FIELD PERIODS

Due to its smaller size, this device might be expected to have enhanced bulk edge coupling when compared to devices we have studied previously. Enhanced bulk edge coupling should be reflected in the Aharonov-Bohm periods and in the size of the discrete jumps in phase.

In the absence of bulk edge coupling, the interference phase will be given by Eqn. 1:

$$\frac{\theta}{2\pi} = e^*_{in}\frac{AB}{\Phi_0} + N_L\frac{\theta_a}{2\pi} \tag{1}$$

B is the magnetic field, A is the interferometer area, e^*_{in} is the quasiparticle charge on the interfering edge state, Φ_0 is the flux quantum, N_L is the number of localized quasiparticles, and θ_a is the anyonic phase. When there is finite bulk edge coupling the phase is modified as given by Eqn. 2 [5]:

$$\frac{\theta}{2\pi} = e^*_{in}\frac{\bar{A}B}{\Phi_0} - \frac{K_{IL}}{K_I}\frac{e^*_{in}}{\Delta\nu}(e^*_{in}N_L + \nu_{in}\frac{\bar{A}B}{\Phi_0} - \bar{q}) + N_L\frac{\theta_a}{2\pi} \tag{2}$$

This modification comes about because there will be variations δA in the area due to the bulk edge coupling. $\phi \equiv \frac{\bar{A}B}{\Phi_0}$ is the flux through the average area $\bar{A}$ (note that $\bar{A}$ does not include modulations δA induced by bulk edge coupling), $\Delta\nu$ is the difference in filling factor between the interfering edge state and the next outer one, ν_{in} is the filling factor corresponding to the interfering edge state, and $\bar{q}$ is the background charge (which may be modified by the gate voltage). This implies that if the number of quasiparticles N_L is kept fixed and the background charge is kept fixed, the device will have an oscillation period given by Eqn. 3:

$$\Delta B = \frac{\Phi_0}{e^*_{in}\bar{A}}(1 - \frac{K_{IL}}{K_I}\frac{\nu_{in}}{\Delta\nu})^{-1} \tag{3}$$

On the other hand, as derived in [4], assuming there is no cost for creating localized quasiparticles (or electrons for integer states), localized charges will be created which make the average change in bulk charge with B zero, and as long as $\frac{K_{IL}}{K_I} < 0.5$ the interference pattern returns to normal Aharonov-Bohm interference (for integer states) with period $\Delta B = \frac{\Phi_0}{e^*_{in}\bar{A}}$ (although at low temperature there will be modulations due to the phase jumps when quasiparticles enter, even for integer states where there is no anyonic phase).

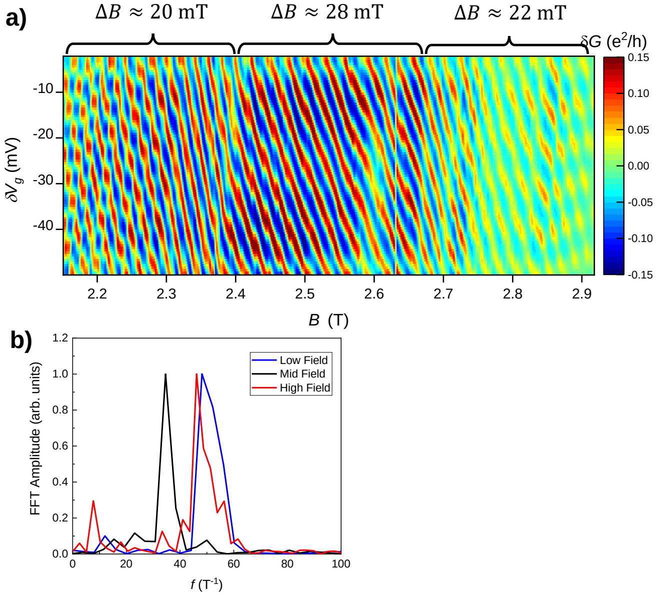

SUPP. FIG. 3. a) Interference across the $\nu = 1$. Though less distinct than at $\nu = 1/3$, the device appears to show three distinct regions with different magnetic field periods. This suggests that there is a central region where μ is in the gap and no localized electrons are created, and regions at low and high field where electrons and holes are periodically added to the device. b) Fourier transforms in the low-field, center, and high-field regions. The peak frequencies are used to extract the magnetic fields listed.

The theory of [7] predicts that the physics of constant ν when μ is in the gap and the bulk is incompressible vs. constant density when the density of states is high

and the bulk is compressible should also apply to integer states. Data from our device at $\nu = 1$ appears to show evidence for this: near the center of the plateau there are AB oscillations with negative slope and a period of ≈ 28 mT, while at lower field and higher field there are oscillations with a somewhat smaller period (≈ 20 mT at low field and ≈ 22 mT at higher field). This is shown in Fig. 3a, and the periods are extracted from Fourier transforms in Fig. 3b. The larger period in the center suggests that the oscillation period is scaled up by the factor $(1 - \frac{K_{IL}}{K_I}\frac{\nu_{in}}{\Delta\nu})^{-1}$ as would be predicted by Eqn. 2 for the case of no change in localized charge, while the smaller periods at high and low field suggest a return to the unscaled period due to high DOS and localized electrons being created as predicted in [7]. This is also supported by the fact that there are periodic modulations visible in the interference pattern in the high and low field regions which are not seen in the center, consistent with shifts in the phase due to periodic changes in localized electron number in those regions (in this case, since $\nu = 1$ is an integer state, these modulations likely occur due to the bulk edge coupling rather than anyonic statistics, and are more prominent in this device due to its smaller size). The ratio of the periods gives $1 - \frac{K_{IL}}{K_I}\frac{\nu_{in}}{\Delta\nu} \approx 0.75$ (for the unscaled period the average of the high and low fields regions of 21 mT is used). For $\nu = 1$ there is a single edge state so $\nu_{in} = \Delta\nu = 1$, so the estimated $\frac{K_{IL}}{K_I}$ is 0.25. Since this value of $\frac{K_{IL}}{K_I}$ is less than 0.5, the device should be in the Aharonov-Bohm regime [4], which is consistent with the fact that the overall behavior is negatively sloped lines of constant phase.

While this shift in behavior is in some ways similar to what is observed at $\nu = 1/3$, a profound difference is that at $\nu = 1$ in the high and low field regions where quasiholes and quasiparticles are being created, the slope of the lines of constant phase becomes steeper (corresponding to a smaller B period), whereas at $v = 1/3$ in the high and low field regions the slope becomes essentially zero as the lines of constant phase become nearly flat. This points to the important difference between anyons and fermions: the effect of removing a $\nu = 1/3$ anyonic quasiparticle (or adding a quasihole) by increasing B is negative shift in phase because $\theta_a = 2\pi/3$, whereas removing a fermionic electron results in a positive shift in phase because the bulk-edge coupling makes the area increase (giving an increase in the Aharonov-Bohm phase).

A similar analysis can be done at $\nu = 1/3$. In between the phase jumps, the oscillation period is ≈ 83 mT, which can be set equal to Eqn. 3 with $e^*_{in} = 1/3$. A is know from the high and low field region oscillation periods at $\nu = 1$ to be $A = \frac{\Phi_0}{\Delta B} \approx 0.2\ \mu\text{m}^2$. Using the oscillation period for the modulations in the high field and low field at $\nu = 1/3$ and assuming these are spaced by Φ_0 yields nearly the same A, which is good evidence that these modulations are indeed due to the anyonic phase of quasiparticles introduced with period Φ_0. Also, since $\nu = 1/3$ has a single edge mode, $\Delta\nu = \nu = 1/3$. Using these values for A and $\Delta\nu$ in Eqn. 3 yields $(1 - \frac{K_{IL}}{K_I}) = 0.76$ and $\frac{K_{IL}}{K_I} = 0.24$. This is quite close to the $\nu = 1$ value. This is reasonable since both states consist of a single edge mode, so a similar charge redistribution is required to change the area of the interference path, resulting in a similar K_{IL}.

SUPPLEMENTAL SECTION 4: SYMMETRY OF POTENTIAL DROP IN FINITE BIAS MEASUREMENTS

For an interferometer the conductance oscillates with the phase, with the conductance varying as $\delta G \propto \cos(\theta)$. When a finite source-drain bias is applied, there are two possibilities to consider: that the potential is applied symmetrically (i.e. the edge state on each side of the device carries an equal amount of the out-of-equilibrium current, with half of the applied bias applied to each edge state) or it is asymmetric (one side carries all or most of the current). For integer states (where $e^* = e$ and $\Delta\nu = 1$) and weak backscattering, when the experiment of measuring differential conductance as a function of gate voltage or magnetic field and source drain bias V_{SD} is performed, the symmetric case results in a checkerboard pattern with $\delta G \propto \cos(\frac{2\pi AB}{\Phi_0})\cos(\frac{LeV_{SD}}{2\hbar v_{edge}})$, whereas for asymmetric potential drop $\delta G \propto \cos(\frac{2\pi AB}{\Phi_0} - \frac{LeV_{SD}}{\hbar v_{edge}})$. In either case the differential conductance should oscillate as a function of V_{SD}. In the symmetric case the product of cosines will result in nodes in the oscillation pattern at $\frac{LeV_{SD}}{2\hbar v_{edge}} = \pi(n + 1/2)$, so that the voltage spacing between nodes $\Delta V_{SD} = \frac{2\pi\hbar v_{edge}}{eL} = \frac{hv_{edge}}{eL} = \frac{\Phi_0\mathcal{E}}{LB}$. In the fully asymmetric case rather than a checkerboard pattern, pajama stripes with constant sloped lines of constant phase would be expected, but the same expression for ΔV_{SD} would hold, except that in this case ΔV_{SD} is the oscillation period as a function of V_{SD}. This gives $E_{sp} = \frac{\delta n_I^2}{2}\Delta V_{SD}$.

The intermediate case is also possible and has been reported in graphene interferometers [8]. Usually in past experiments in GaAs a checkerboard pattern consistent with symmetric potential drop has been seen [9, 10]. Interestingly, in this device when measuring the differential conductance the symmetry of the applied bias depends on the meausurement circuit and how the potential of the screening wells is set relative to the source and drain contacts. To simplify interpretation we focus on measurements where the potential drop appears to be close to symmetric in the main text.

In our device, the screening wells are isolated from all of the Ohmic contacts except for one, which prevents any current flowing through the screening wells but ensures that the screening wells are at fixed potential. The single non-isolated ohmic is usually used as a grounding

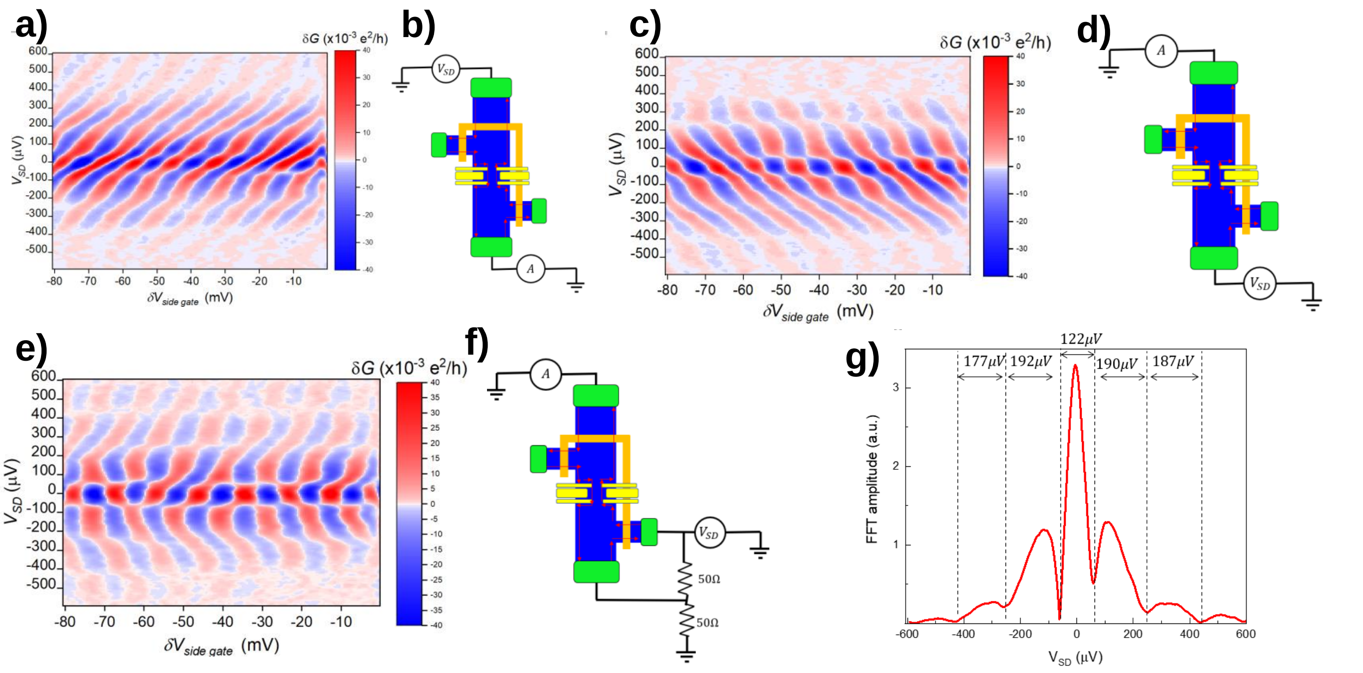

SUPP. FIG. 4. a) Differential conductance measurements at $\nu = 1/3$ $B = 7.4$ T. The overall positive slope suggests that the bulk 2DEG tends to come closer to the potential of the drain contact. b) Schematic showing the cirucit used for the data in a). Green represents the Ohmic contacts, blue represents the mesa and 2DEG, yellow represents the interferometer gates, and orange represents the gate used to isolate the top SW from the Ohmics (there is also an additional gate, not shown, on the reverse side of the chip which isolates the bottom SW from the Ohmics). The bottom Ohmic is not subtended by these gates and thus the SWs equilibrate with this bottom Ohmic. In this circuit setup the bottom contact is used as the drain, with the applied V_{SD} applied to the opposite side of the mesa. c)Differential conductance at $\nu = 1/3$ with the source and drain contacts swapped, with circuit shown in d). e) Differential conductance with non-isolated Ohmic fixed at $V_{SD}/2$, using the circuit shown in f). g) Oscillation amplitude vs. V_{SD}. The spacing between nodes (which appear as minima in the plot) are indicated.

contact; differential conductance data using this configuration is shown in Supp. Fig. 4a, and the circuit is illustrated in Supp. Fig. 4b. It is apparent that there is an overall positive slope to the data, although the data also has some of the character of the checkerboard pattern expected for symmetric potential drop. This suggests that potential in the bulk of the 2DEG inside the interferometer is close to the drain potential rather than being symmetric between the source and drain potential. If the source and drain contacts are switched so that the Ohmic connected to the screening wells is used as the source contact, the slope of the data switches, illustrated in Supp. Fig. 4c and d, indicating that the bulk of the 2DEG is coming close to the potential of the source contact. This indicates that the potential of the screening wells tends to set the electrostatic potential in the bulk of the 2DEG, which is to be expected due to their short setback from the main quantum well.

To achieve the symmetric case, we have implemented a unique biasing scheme in which the non-isolated Ohmic is fixed at 1/2 of the applied V_{SD} (this can be done without directly affecting the current across the device by applied V_{SD} to a downstream Ohmic). The resulting data is shown in Supp. Fig. 4e and the schematic is shown in Supp. Fig. 4f. While the data is fairly symmetric, there is some positive slope behavior when V_{SD} is positive and negative slope when V_{SD} is negative, suggesting that the bulk tends to reach a potential which is somewhat closer to the higher-energy edge state. A similar pattern of different symmetries depending on the source-drain contact configurations occurs at $\nu = 1$, although it is somewhat less pronounced. To simplify interpretation of the data we have analyzed data sets which have this nearly symmetric behavior, which allows the method of extracting velocity from node spacing to be applied as in previous works. The DC current measurements also use the symmetric biasing configuration.

SUPPLEMENTAL SECTION 5: FINITE-BIAS MEASUREMENTS AT ELEVATED TEMPERATURE

As discussed in the main text, at low temperature the finite bias current measurements at $\nu = 1/3$ exhibit a non-uniform node spacing in agreement with the predictions of [3]. We have also measured oscillations at an elevated mixing chamber temperature of 130 mK, shown

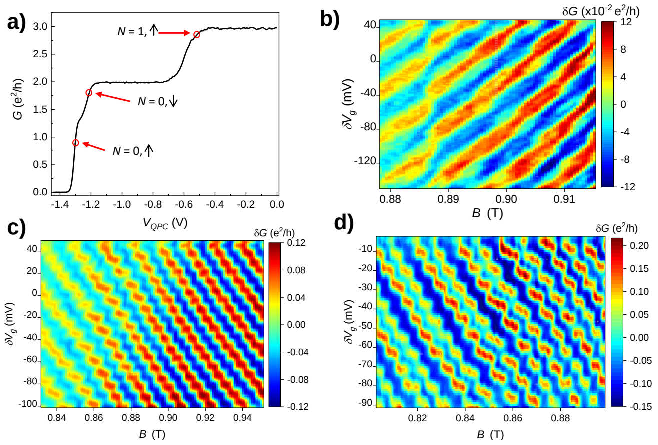

SUPP. FIG. 5. a) QPC sweep at $\nu = 3$. There are clear conductance plateau at $G = 3\frac{e^2}{h}$ and $G = 2\frac{e^2}{h}$; there is not a clear $G = 1 \times \frac{e^2}{h}$ plateau, likely because the spin gap is too small compared to the cylotron gap, so the two spins of the N = 0 Landau level cannot be completely independently transmitted. However, there is a wiggle in the data that may delineate between primarily backscattering the inner (spin up) N = 0 edge state and primarily backscattering the outer (spin down) N = 0 edge state. Red circles indicate the QPC points where the device is likely primarily partially reflecting a single edge state. b) Repeated from the main text, interference of the innermost edge state corresponding to N = 1, spin up (the corresponding QPC operating point is shown in a). As discussed in the main text, the positive slope indicates Coulomb-dominated behavior, consistent with the value of $\frac{K_{IL}}{K_I} = 0.65 > 0.5$ discussed in the main text. c) Interference of the middle edge state, with QPC operating point also shown in a). The overall negative slope to the data indicates Aharonov-Bohm regime behavior, suggesting that a steeper confining potential towards the outer edge of the sample results in a larger K_I. Modulations in the pattern are visible, indicating that effects of bulk-edge coupling are still present. d) Interference of the outermost edge state, with operating point indicated in a). In this case the device exhibits oscillations with approximately half the expected Aharonov-Bohm periods, consistent with previous observations of period-halving for the outermost edge mode when an inner mode is present (and fully reflected). This has been explained by an inter-edge coupling [6], which is highly plausible because the the two N = 0 edge states are very close together, and also possibly by electron pairing (note the lack of a clear e^2/h plateau in a distinguishing the separate spin edge states supports these two edge states being close together). Clear modulations in the interference pattern are visible suggesting that there may be complicated interplay between bulk-edge coupling and edge-edge coupling.

in Supp. Fig. 6. At this elevated temperature the innermost node (corresponding to the minima in the FFT amplitude) moves outward to higher V_{SD} compared to the low-temperature measurement; this comparison is shown in Supp. Fig. 6b. The inner nodes move from approximately -166 μV and +166 μV at 10 mK to -178 μV and +185 μV at 130 mK. The outer node spacing is approximately 197 μV, so at 130 mK the node spacing has become nearly uniform.

It is also noticeable that at small V_{SD}, the oscillation amplitude is much larger at 10 mK mixing chamber temperature than at 130 mK, consistent with thermal dephasing. At elevated V_{SD}, however, this difference is less pronounced. This suggests that large V_{SD} causes significant heating of the electrons in the device so that the electron temperature is above the mixing chamber temperature. This might explain why the ratio of inner node spacing is larger than the value of $\frac{1+g}{2} = 2/3$ expected by [3] at low temperature, since the applied bias may already cause significant heating and a partial shift towards the high-temperature limit of uniform spacing.

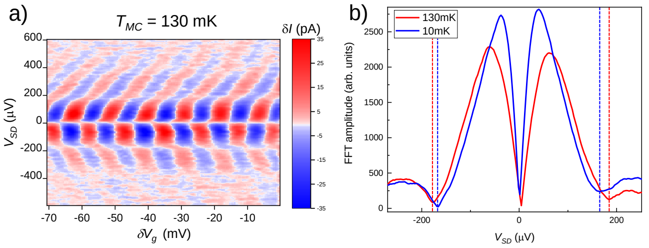

SUPP. FIG. 6. a) DC current oscillations at $\nu = 1/3$, $B = 7.4$ T as a function of side gate voltage δV_g and V_{SD}. b) Oscillation amplitude versus V_{SD} at 10 mK and 130 mK. Dashed lines indicate the positions of the minimum in the oscillations, which correspond to the innermost node. At 130 mK these nodes occur at approximately -178 μV and +185 μV, and at 10 mK they occur at -166 μV and +166 μV. The fact that these inner nodes move to larger V_{SD} and approach the outer node spacing of $\approx$ 195 μV at elevated temperatures is consistent with the expected Luttinger-liquid behavior from the model of [3].

SUPPLEMENTAL SECTION 6: CALCULATING PHASE BY FOURIER TRANSFORM

In order to accurately calculate the values of the phase jumps that occur at $\nu = 1/3$, we have employed a different method in which the phases are extracted from a Fourier transform of the conductance data. Fourier transforms are used to find the value of the phase θ at each value of magnetic field. These FFTs are taken along cuts of conductance that are parallel to the discrete jumps in phase so that they do not cross the discrete jumps; this enables the discrete jumps to be made as sharp as possible in plots of θ versus B. The slope of these cuts is 0.4 V/T, as illustrated in Supp. Fig. 7a.

The phases extracted from the FFT, plotted in Supp. Fig. 7b, are defined from $-\pi$ to π. To remove the discontinuities that occur when crossing this range, the data is shifted up when there is a crossover from $-\pi$ to $+\pi$, as illustrated by the arrows in Supp. Fig. 7b. The resulting phases are plotted in Supp. Fig. 7c.

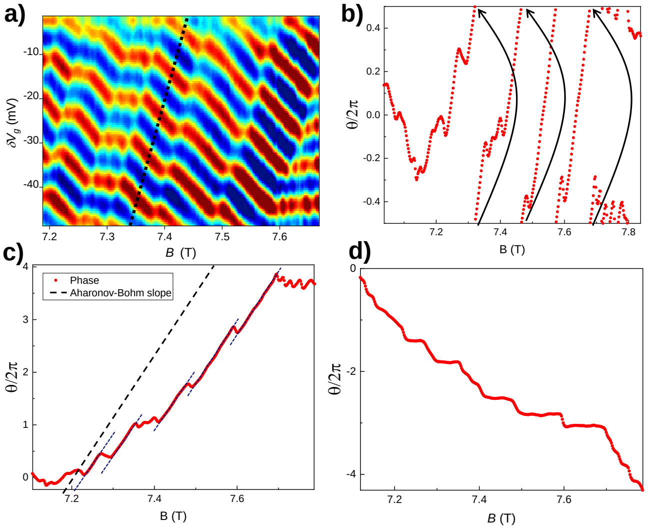

SUPP. FIG. 7. a) Interference data at $\nu = 1/3$; this is a subset of the data shown in the main text. b) Phases extracted from Fourier transforms of the data in a). The phase is evaluated at the peak frequency which corresponds to the Aharonov-Bohm oscillation frequency c)Phase after shifting the phase by 2π when it crosses from $+\pi$ to $-\pi$ to avoid these discontinuities. The black dashed line indicates the constant Aharonov-Bohm slope, and the blue dashed lines indicate that this slope is consistent in the regions between jumps. d) Phases with the Aharonov-Bohm slope subtracted to isolate the contribution from the discrete phase jumps.

In the central region where the discrete jumps in phase are mostly well isolated from each other, the phase primarily evolves due to the Aharonov-Bohm effect, which gives a constant phase evolution $\frac{d\theta}{dB} = \frac{2\pi e^* A}{\Phi_0}(1 - \frac{K_{IL}}{K_I}\frac{\nu_{in}}{\Delta\nu})$; here the effect of finite bulk-edge interaction is included. This slope can be found by calculating the slope in between the discrete jumps, resulting in an Aharonov-Bohm slope of ≈ 0.012 T^{-1} (corresponding to a period of 83 mT). This enables extraction of $\frac{K_{IL}}{K_I}$ as discussed in the main text. This slope is shown by the dashed line in the figure, and is consistent across the different regions between the discrete jumps, as expected. In order to isolate the phase contributions from the anyonic statistics found in the discrete jumps, this Aharonov-Bohm slope is subtracted off, with the resulting phase shown in Fig. 7d; this is the same data shown in the main text. Plateaus occur corresponding the regions between the discrete jumps, and the value of the discrete jumps can be computed by the difference in phase from one plateau to another.

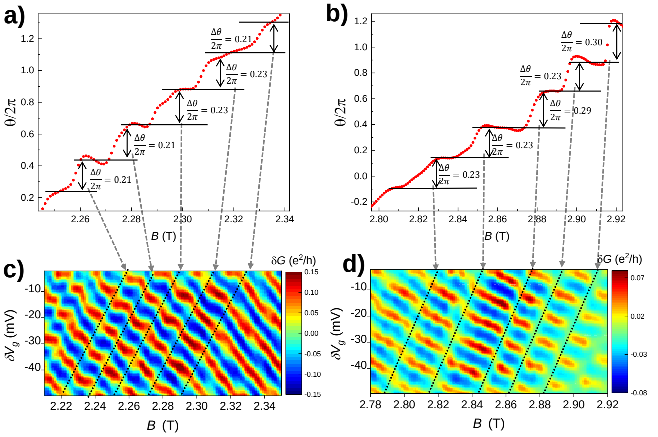

SUPP. FIG. 8. Analysis of discrete phase jumps at $\nu = 1$. a) values of the phase extracted from FFT for the low field region at $\nu = 1$ and b) for the high-field region. c) Conductance data indicating where the discrete jumps in phase occur in the high low field region and d) in the high field region. In the low field data the average value of the phase jump is $\frac{\Delta\theta}{2\pi} = 0.22 \pm 0.02$, while in the high field data it is $\frac{\Delta\theta}{2\pi} = 0.26 \pm 0.04$, which comports with the values of $\frac{K_{IL}}{K_I}$ extracted at $\nu = 1$ in the main text.

SUPPLEMENTAL SECTION 7: ANALYSIS OF DISCRETE PHASE JUMPS DUE TO BULK-EDGE COUPLING IN THE INTEGER REGIME

As discussed in the main text and given by Eqn. 3, in the integer quantum Hall regime discrete jumps in phase can occur when the localized electron number changes due to bulk-edge coupling. The value of these phase jumps will be given by $\frac{\Delta\theta}{2\pi} = \frac{e^{*2}}{\Delta\nu}\frac{K_{IL}}{K_I} = \frac{K_{IL}}{K_I}$. Such discrete jumps would be expected to be visible at integer states when the bulk is compressible, such as in the high and low field regions at $\nu = 1$. In Supp. Fig. 8 we have re-plotted data from $\nu = 1$ in the high and low field regions, showing that jumps in phase can indeed be seen in some ranges of magnetic field.

Using the same method described in Supp. Section 6, we have extracted the phase by FFT and removed the continuously-varying Aharonov-Bohm contribution to isolate the phase due to the discrete jumps, plotted in Supp. Fig. 8a (low field) and b (high field). The corresponding regions from the data where the jumps occur are labeled in Supp. Fig. 8c and d. A significant qualitative difference between these jumps at integer filling and those at $\nu = 1/3$ is that the jumps at $\nu = 1/3$ have a negative value, while these at $\nu = 1$ have a positive value for $\Delta\theta$, consistent with being due to bulk-edge coupling rather than anyonic statistics. The discrete jumps have an average value of $\frac{\Delta\theta}{2\pi} = 0.22 \pm 0.02$ in the low-field region and $\frac{\Delta\theta}{2\pi} = 0.26 \pm 0.04$ in the high field region, which is close to what would be expected given the value of

$\frac{K_{IL}}{K_I} = 0.24$ extracted from the ratio of magnetic field periods, and slightly lower (but still in reasonable agreement with) the value of $\frac{K_{IL}}{K_I} = 0.31$ extracted from finite bias measurements. It is noteworthy that the phase jumps at $\nu = 1$ blur together somewhat, which may be due to the relatively small separation in terms of magnetic field between them, and makes the plateaus in phase not completely flat.

SUPPLEMENTARY MATERIAL REFERENCES

[1] Roosli, M. P. *et al.* Observation of quantum Hall interferometer phase jumps due to a change in the number of localized bulk quasiparticles. *Phys. Rev. B* **101**, 125302 (2020)

[2] M. P. Roosli *et al.* Fractional Coulomb blockade for quasiparticle tunneling between edge channels. *Science Advances*, **7**, eabf5547 (2021)

[3] Chamon, C. de C., Freed, D. E., Kivelson, S. A., Sondhi, S. L., & Wen, X. G. Two point-contact interferometer for quantum Hall systems. *Phys. Rev. B* **55**, 2331 (1997)

[4] Halperin, B. I., Stern, A., Neder, I., & Rosenow, B. Theory of the Fabry-Perot quantum Hall interferometer. *Phys. Rev. B* **83**, 155440 (2011)

[5] von Keyserlingk, C. W., Simon, S. H., & Rosenow, B. Enhanced Bulk-Edge Coulomb Coupling in Fractional Fabry-Perot Interferometers. *Phys. Rev. Lett.* **115**, 126807 (2015)

[6] G. A. Frigeri, D. D. Scherer, & B. Rosenow. Sub-periods and apparent pairing in integer quantum Hall interferometers. *EPL* **126**, 67007 (2019)

[7] Rosenow, B., & Stern, A. Flux Superperiods and Periodicity Transitions in Quantum Hall Interferometers. *Phys. Rev. Lett.* **124**, 106805 (2020)

[8] Deprez, C. *et al.* A tunable Fabry-P´erot quantum Hall interferometer in graphene. *Nature Nanotechnology* (2021)

[9] McClure, D. T., *et al.* Edge-State Velocity and Coherence in a Quantum Hall Fabry-Perot Interferometer. *Phys. Rev. Lett.* **103**, 206806 (2009)

[10] Nakamura, J. *et al.* Aharonov-Bohm interference of fractional quantum Hall edge modes. *Nat. Phys.* **15**, 563-569 (2019)

[11] D. E. Feldman and B. Halperin. Fractional charge and fractional statistics in the quantum Hall effects. *arxiv* https://arxiv.org/abs/2102.08998 (2021)
